# Study of C-reactive Protein Levels in Hypertensive Disorders of Pregnancy

**DOI:** 10.7759/cureus.66946

**Published:** 2024-08-15

**Authors:** Sangeeta B Singh, Shikhaa Mahajan, Krishnanand PS, Divya Mangla

**Affiliations:** 1 Biochemistry, Shaheed Hasan Khan Mewati Government Medical College, Nuh, IND; 2 Obstetrics and Gynecology, Shaheed Hasan Khan Mewati Government Medical College, Nuh, IND

**Keywords:** preeclampsia, hypertensive disorders of pregnancy (hdp), inflammation, hypertension, c-reactive protein level

## Abstract

Introduction

Globally, one of the main causes of maternal and perinatal deaths is hypertensive disorders of pregnancy (HDP). Preeclampsia is a pregnancy-specific syndrome characterized by high blood pressure and proteinuria after 20-week gestation. Women who develop preeclampsia are at increased risk for the development of many systemic complications.

Materials and methods

A cross-sectional study was conducted in the Department of Biochemistry, SHKM, Nuh, Haryana in collaboration with the Department of Obstetrics & Gynecology. 200 study subjects; Group 1: cases - 100 HDP, Group 2: controls - 100 age-matched normotensive pregnant women. The specimen of 3 mL venous blood was collected under aseptic precautions. The data was evaluated using statistical evaluation tools.

Result

Out of 200 subjects, mean C-reactive protein (CRP) levels were normal in three cases and 35 controls. Minor elevation of CRP was observed in 11 cases and 29 controls (very significant). Moderate elevation of CRP was observed in 61 cases and 36 controls (highly significant). Marked elevation of CRP was observed in 25 cases.

Mean CRP levels increased from preeclampsia without severe features (3.9 mg/dL), preeclampsia with severe features (5.2 mg/dL), and eclampsia (12.7 mg/dL) when compared with the respective control group (1.1 mg/dL, 1.16 mg/dL, and 1.2 mg/dL).

Conclusion

There is a highly significant association between CRP levels and HDP. Additionally, one useful indicator of the severity of preeclampsia is CRP, a measure of systemic inflammation. Elevated CRP levels in mild PE can be used as an indicator of the progress of the disease and early prevention can be done.

## Introduction

The most prevalent medical condition that arises during pregnancy is hypertension, which complicates 2% to 3% of pregnancies. Four categories are recommended by the American College of Obstetricians and Gynecologists for high blood pressure in pregnancy for hypertensive disorders of pregnancy (HDP): preeclampsia without severe features, preeclampsia with severe features, and eclampsia and gestational hypertension [1].

Preeclampsia is a pregnancy-specific condition that affects up to 8% of pregnant women and is characterized by elevated blood pressure and proteinuria after 20 weeks of gestation. It is affecting different systems of the body with little known reason. Preeclampsia is defined as systolic blood pressure of the mother of more than 140 mmHg and diastolic blood pressure of more than 90 mmHg at two different times, which are divided by at least six hours and proteinuria greater than 300 mg in one day (24 hours) period after 20 weeks of gestation [1].

Eclampsia is the development of convulsions in a pre-existing preeclampsia or it may appear suddenly in a patient with slightly elevated blood pressure and no proteinuria [1].

Preeclampsia and eclampsia advance in 4.6% and 1.4% of pregnancies worldwide, respectively. Preeclampsia incidence varies from 1.8% to 16.7% in developing countries, while it is approximately 3% in developed countries [2].

The villous cytotrophoblast enters the uterus's myometrium in healthy placental growth and remodels the spiral arteries, increasing their capacitance and decreasing their resistance. The shallow cytotrophoblast invasion in preeclampsia causes decreased placental perfusion and placental insufficiency, which in turn causes hypoxia and endothelial dysfunction. Therefore, the cause of preeclampsia could be abnormal placentation or abnormal decidualization [3].

Hepatic cells from the liver release CRP, an acute phase protein, in exposure to proinflammatory cytokines. In preeclampsia, it is a sensitive indicator of inflammation resulting in endothelial dysfunction. Its sensitivity and specificity in predicting PE have been documented in numerous studies [4].

One objective and sensitive marker of the body's total inflammatory activity is CRP, which has been proposed as a potential sign of preeclampsia [5].

## Materials and methods

The present cross-sectional study was conducted in the Department of Biochemistry, SHKM GMC, Nuh, Haryana in collaboration with the Department of Obstetrics and Gynecology.

Pregnant women aged 18 to 45 newly diagnosed with hypertension after 20 weeks of gestation with single intrauterine pregnancy were included in the study.

Critically ill, bedridden patients, females with twin pregnancies, females of age above 45 years, patients with chronic illnesses like chronic hypertension, diabetes mellitus, heart disease, thyroid disorders, liver diseases, morbid obesity, gestational diabetes, gout, acute and chronic kidney disease, and any other chronic illnesses, and taking any medications for these were excluded from the study. A total of 200 study subjects were recruited for the study, and they were further divided into two groups. Group 1: cases - 100 diagnosed patients of HDP were selected and were further subdivided into three groups: (a) preeclampsia without severe features, (b) preeclampsia with severe features, and (c) eclampsia. Group 2: controls - 100 age-matched normotensive pregnant women.

A history was taken as per the attached study proforma and a blood sample was collected for the routine and special investigations. After informed consent, 6 mL of venous blood was drawn from the antecubital vein in red vacutainers while maintaining aseptic conditions. The sample was sent right away to the lab, where it was centrifuged; the serum was separated, and it was examined. After clotting, serum was separated by centrifugation for five minutes at 3000 revolutions per minute (RPM). Routine investigations were carried out on the same day and serum was stored at -20˚C for further analysis of CRP.

In our study, normal CRP levels were defined as less than 0.3 mg/dL, minor elevation of CRP was defined as 0.3 to 1.0 mg/dL, moderate elevation of CRP was defined as 1.0 to 10.0 mg/dL, and marked elevation of CRP was defined as more than 10.0 mg/dL [6].

CRP was measured in the biochemistry laboratory using a Cobas 6000 auto analyzer. The measuring range for CRP was 3.0‑400 mg/L or 0.30‑40.0 mg/dL. This machine mixes the plasma sample with CRP antibody latex reagent in a reaction chamber after diluting it with HEPES (4-(2-hydroxyethyl)-1-piperazineethanesulfonic acid) buffer. The CRP antibody on the latex particle binds to the CRP in the diluted plasma. The amount of agglutination is related to the altered absorbance measured at 525 nm and 625 nm, which is used for calculating the concentration of CRP [7].

## Results

Out of 200 subjects, mean CRP levels were observed to be normal in three cases (0.16 mg/dL) and 35 controls (0.11 mg/dL). Mild elevation of mean CRP was observed in 11 cases (0.45 mg/dL) and 29 controls (0.61 mg/dL) with a p-value of 0.01 (very significant). Moderate elevation of mean CRP was observed in 61 cases (4.64 mg/dL) and 36 controls (2.59 mg/dL) with p-value <0.001 (highly significant). Marked elevation of mean CRP was observed in 25 cases (15.45 mg/dL). The results are given in Table 1 and Table 2.

**Table 1:** Number of patients in different categories of CRP CRP, C-reactive protein

CRP Level (Normal: 0-0.3 mg/dL)	Cases	Controls
Normal (<0.3 mg/dL)	3	35
Mildly elevated (0.3-1 mg/dL)	11	29
Moderately elevated (1-10 mg/dL)	61	36
Markedly elevated (>10 mg/dL)	25	0

**Table 2:** Mean CRP levels and their significance CRP, C-reactive protein

CRP levels	Cases	Controls	P-value	Significance
Normal (0.3 mg/dL)	0.16	0.11	0.12	Not significant
Mildly elevated (0.3-1 mg/dL)	0.45	0.61	0.01	Very significant
Moderately elevated (1-10 mg/dL)	4.64	2.59	<0.001	Highly significant
Markedly elevated (>10 mg/dL)	15.45	0	Nil	Nil

The mean CRP level was 3.9 mg/dL in mild PE compared with the control group with a mean value of 1.1 mg/dL having a p-value <0.001, 5.2 mg/dL in severe PE compared with the control group with a mean value of 1.16 mg/dL, having p-value <0.001, and 12.7 mg/dL in eclampsia compared with the control group with a mean value 1.2 mg/dL having p-value <0.001, which showed highly significant correlation. The results are given in Table 3.

**Table 3:** Mean CRP levels in different categories of HDP CRP, C-reactive protein; HDP, hypertensive disorders of pregnancy

Severity of HDP	No. of patients	Mean of CRP in cases (mg/dL)	Mean of CRP in controls (mg/dL)	P-value	Significance
PE without severe features	37	3.9	1.1	<0.001	Highly significant
PE with severe features	37	5.2	1.16	<0.001	Highly significant
Eclampsia	26	12.7	1.2	<0.001	Highly significant

## Discussion

Regarding the mother's age, gestational age, and body mass index, there were no significant variations between the patient and control groups in our study. The study revealed that patients' CRP levels were higher than those of the control group. There is a direct correlation between the severity of PE and this increase.

The current study's observation of a significantly higher level of CRP in the case group compared to the control group (P<0.001) is consistent with numerous prior studies where authors proposed that CRP is a promising biochemical marker that can indicate the severity of preeclampsia [8].

In a study by Savvidou et al., 26 cases of pregnancies that were normal, 26 cases of preeclampsia that were mild, and 26 cases of preeclampsia that were severe in the third trimester of pregnancy reported that plasma CRP levels in mild and severe preeclampsia patients were markedly higher than that of normal third trimester pregnant women. The study found higher levels of CRP in cases compared with that of controls and the presence of a good correlation between CRP and preeclampsia [9].

In our study, the severity of preeclampsia increased with increasing levels of CRP. The results were consistent with the findings of Chunfang et al., which also stated that as CRP levels climbed, the risk of preeclampsia increased as well. The study was a prospective, nested, case-control study design where they evaluated serum levels of CRP concentrations using the competitive ELISA method in 506 women who maintained normotension throughout their pregnancy and 60 women who subsequently had preeclampsia. A distinct predictor and potential risk factor for preeclampsia in women, the elevated CRP value, was found to have a strong correlation with pre-pregnancy obesity [10].

In a study by Ali et al., to look for differences in CRP levels, 100 singleton pregnant women were split into 50 patients with preeclampsia and 50 healthy pregnant women as the control group. The study found that CRP was significantly higher in preeclampsia groups in comparison to the control group and the increment was directly correlated with the severity of preeclampsia. The study also suggested that early diagnosis of preeclampsia is possible with the levels of CRP [11]. This study's results were in parallel with the findings of the present study.

Additionally, in a study by Renu et al., in 150 cases of preeclampsia and 50 control population, the study found that the mean value of CRP in the case group was 24.8 mg/dL and that of the control group was 1.2 mg/dL, which proved to be highly significant. This study concluded that by monitoring the value of CRP, it may be able to predict the progress of preeclampsia in pregnant women [12]. The results and conclusion of the study are similar to the results of the present study.

Another study by Tjoa et al. measured CRP levels in 107 women visiting obstetrics and gynecology departments for antenatal checkups and found that the mean levels of CRP were significantly higher in females who developed preeclampsia in the later stages of pregnancy. The study suggested that early CRP testing can be used as a marker for predicting the development of preeclampsia and complications like intrauterine growth restriction [13]. The findings of the study are in agreement with the present study.

A study by Shetty et al. included 200 singleton pregnant subjects whose CRP levels were recorded and followed up on maternal and fetal outcomes. The study found that in severe preeclampsia, there are increased levels of CRP and decreased levels of platelets. The study suggested that the prognosis of the pregnant female outcome may be related to the levels of CRP in serum. The suggestions of the study are in parallel with the present study [14].

In a study by Paternoster et al. conducted on 322 pregnant women from 24 weeks to 32 weeks, the study found that the mean CRP levels tend to increase with the complications of pregnancy such as decreased birth weight and development of eclampsia. The study suggested that CRP levels can be correlated with the process of development of preeclampsia [15].

## Conclusions

In the present study, there is a highly significant correlation between CRP levels and HDP. CRP, being a systemic inflammatory response marker, can be used as a valuable indicator for the progression of severity of preeclampsia, which is among the most problematic diseases of obstetrics. It is important to educate all women of reproductive age groups regarding risk factors and complications of preeclampsia. Elevated CRP levels in preeclampsia can be used as an indicator of progress of the disease and early prevention can be done.
